# Numbers and distribution of the Great Cormorant in Iceland: Limitation at the regional and metapopulation level

**DOI:** 10.1002/ece3.5028

**Published:** 2019-03-12

**Authors:** Arnthor Gardarsson, Jón Einar Jónsson

**Affiliations:** ^1^ Institute of Biological and Environmental Sciences University of Iceland Reykjavik Iceland; ^2^ Research Centre at Snæfellsnes University of Iceland Stykkishólmur Iceland

**Keywords:** climate, disturbance, fecundity, food, regional and total population limitation, seabird, survival

## Abstract

1. We studied a metapopulation of great cormorant *(Phalacrocorax carbo) *in Iceland, using complete aerial censuses of nests in 25 years during 1975–2015. Age composition was estimated in 1998–2014 by ground surveys in September and February. Brood size was estimated from aerial photographs in 2007–2015.
2. Weather, food, breeding habitat, and density were considered as explanatory variables when examining numerical and distributional changes in the cormorant metapopulation.
3. In 1975–1990 total nest numbers changed little, very low numbers about 1992 were followed by an annual increase of 3.5% in 1994–2015. Total nest numbers were positively correlated with estimates of spawning stocks of cod and saithe and inversely related to the subpolar gyre index (SPG‐I).
4. During the increase in 1994–2015, average colony size at first increased and then declined. Habitat use also changed: the proportion of nests on small rocky islets (skerries) at first declined, from 69% to 44% in 1995–2003 and then increased again to about 58% in 2012–2014. Habitat changes were probably a response to changed patterns of human disturbance.
5. Breeding density, as nests per km^2^ sea <20 m deep, was rather uniform among five defined regions in 1975–1996. Thereafter, densities became much higher in two sheltered regions with kelp forests and in one mostly exposed region. A second exposed region remained low and in the third nest numbers declined markedly. Thus, carrying capacity was higher in sheltered regions where cormorant breeding had historically been depressed by human disturbance.
6. Brood size varied little among regions but declined with the years from about 2.5 to 1.8.
7. The proportion of juveniles in September (fecundity) declined in 1998–2015 from over 0.4 to 0.3 and was inversely correlated with year and nest numbers, if outlier years were excluded, suggesting resource limitation. Survival of juvenile cormorants in September–February was estimated at 0.471 ± 0.066 *SE*. Commercial fish stocks and climate indices were not correlated with the proportion of juveniles.
8. Annual survival of adults (breeding and nonbreeding) was estimated from nest counts and age composition 1999–2014, as 0.850 ± 0.026 *SE* and showed no trend in 1998–2014.
9. We conclude that the metapopulation of cormorants in Iceland was resource‐limited at two levels: fecundity at the regional and winter survival at the total level.

## 1 INTRODUCTION

Birds are generally more visible than other vertebrates and so can be counted with some accuracy, at least during the breeding season (McKellar, Marra, Boag, & Ratcliffe, 2014). However, most birds are relatively mobile and spend much time outside a study area, during migration or because of central place foraging. These features complicate the interpretation of complex processes such as population regulation or limitation. Another challenge with local population studies is the question of demarcation of the study population in relation to a population or metapopulation over a wider range (Hanski, 1999).

Ashmole (1963) initially hypothesized that food availability within a foraging radius of breeding colonies limited seabird populations. Ashmole's hypothesis was at first restricted to tropical pelagic seabirds but has since been extended to Arctic seabirds and has found support in both empirical work and modelling (Elliott et al., 2009; Hemerik, Van Opheusden, & Ydenberg, 2014). Thus, resource availability often determines breeding distribution and nest location often is a compromize between safety and access to food resources. Colonial seabirds generally are central place foragers, with the breeding colony serving as the central placement to optimize access to foraging grounds (Burke & Montevecchi, 2009; Christensen‐Dalsgaard, May, & Lorentsen, 2018; Elliott et al., 2009; Sandvik et al., 2016; Shoji et al., 2015; Weimerskirch, 2007). Upon population growth, colonies fill up with more nesters and reach their capacity as all nest sites become occupied, which requires recruits to seek out new territories or occupy suboptimal nest sites at the original colony (Pyk, Weston, Bunce, & Norman, 2013). Ideally, population regulation should be studied for a number of consecutive years, with regard to both local breeding colonies or populations and the total or flyway population.

Ashmole's (1963) halo may well be valid for population limitation on the restricted scale of foraging radius from a seabird colony. Local resources may indeed apply as a limiting factor to any sum of colonies but on a larger scale, the population in question may be limited by conditions away from the breeding colonies in space or time. More recently, workers on bird and mammal populations have begun to examine population limitation in open systems where breeding, staging or wintering sites of migratory populations may be important (Gardarsson & Einarsson, 1994, 1997; Gill et al., 2001; Sherry & Holmes, 1996). This naturally leads to questions of spatial as well as temporal scale.

Bird populations all over the world have responded to climate change, either by changes in numbers or altered migration or nest initiation dates (Sæther, Sutherland, & Engen, 2004; Stephens et al., 2016). Climate change has been implicated in seabird studies but climate indicators have varying relationships with indices for seabird species, for instance the North‐Atlantic Oscillation index (NAO) and similar indices are either positively or negatively related to timing of breeding, or have seemingly no effect (Moe et al., 2009; Wanless, Frederiksen, Walton, & Harris, 2009). Food often limits breeding birds (Martin, 1987; Newton, 1980) but few studies have considered food and climate variables simultaneously. In Norway, researchers found that fish abundance was relatively more important for European shags (*Phalacrocorax aristotelis)* than climate variables (Bustnes, Anker‐Nilssen, Erikstad, Lorentsen, & Systad, 2013; Lorentsen, Anker‐Nilssen, Erikstad, & Røv, 2015). In Eurasian wigeon at Mývatn, Iceland, the production of young was positively related to food abundance and negatively to snaps of cold and wet weather (Gardarsson & Einarsson, 1997).

In addition to climate change, that is, warming trends, there have been changes in the oceanic currents within the Northern Atlantic Ocean in recent decades which have affected flow of nutrients (Hátún et al., 2016). The subpolar gyre index (SPG‐I) was colinear to the NAO until 1995 but the two became de‐coupled in 1995 and the SPG‐I has shown consistently negative values after that event (Berx & Payne, 2017). The subpolar gyre affects flow of nutrients within the ocean, including phosphate, nitrate and silicates (Hátún et al., 2017, 2016; Johnson, Inall, & Häkkinen, 2013). SPG‐I is highly correlated to body size and body mass in Icelandic arctic foxes (*Vulpes lagopus*), presumably because the SPG‐regulated ocean forces affect food availability to the foxes, particularly in coastal habitats in west Iceland where seabirds are an important part of the fox's diet (Yom‐Tov, Hersteinsson, Yom‐Tov, & Geffen, 2017).

Seabirds often occur as groups of colonies that form metapopulations over large areas but only exceptionally have these been subjected to coordinated demographic studies (Oro & Ruxton, 2001). It would appear that an ideal study population would be a whole metapopulation where the responses of many local populations can be examined and interpreted in relation to the whole.

**Figure 1:**
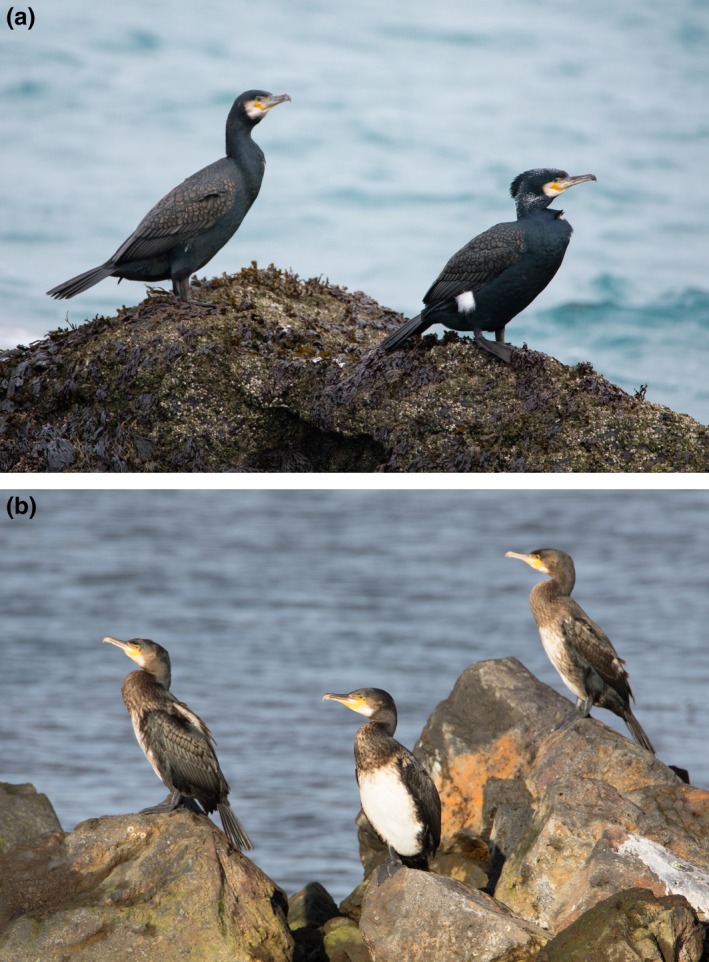
Cormorant *Phalacrocorax carbo carbo* in Iceland. (a) Top panel: adult cormorants can be separated into two groups in February, adult nonbreeders (all black, without filoplumes, left), and full‐plumaged adult breeders (white thigh patches, white filoplumes on head and neck, nuchal crest, right); in September these represent one age group as adults. (b) Bottom panel: in September and February, juveniles (pied brown with a variable amount of white below on chest and belly, all three birds shown) were distinguished from adults. Photos by Erling Ólafsson

We present a long‐term study of an isolated, colonially nesting seabird population, great cormorants of the Atlantic subspecies *(Phalacrocorax carbo carbo)* breeding in Iceland. We chose to study this metapopulation because (a) it is relatively small and isolated from other metapopulations of the same species, the nearest of which are found in Scotland and Greenland, 800 and 1,200 km away, respectively, (b) nests are in small but conspicuous colonies and the entire breeding population can be censused accurately from the air, (c) it can be surveyed in coastal waters throughout the year, (d) age categories can be distinguished in the field (Figure 1), making it possible to observe some demographic features with relatively small effort.

By estimating breeding numbers, fecundity, and distribution on a regional and total scale we hope to gain insight into features, such as climate, food and disturbance, likely to influence the demography of local breeding populations and how these conform to the metapopulation. Like many other seabird populations, our study population has obviously been affected by a long history of human exploitation and disturbance, an influence that became more evident during the course of this study.

## 2 METHODS

### 2.1 Study population

**Figure 2:**
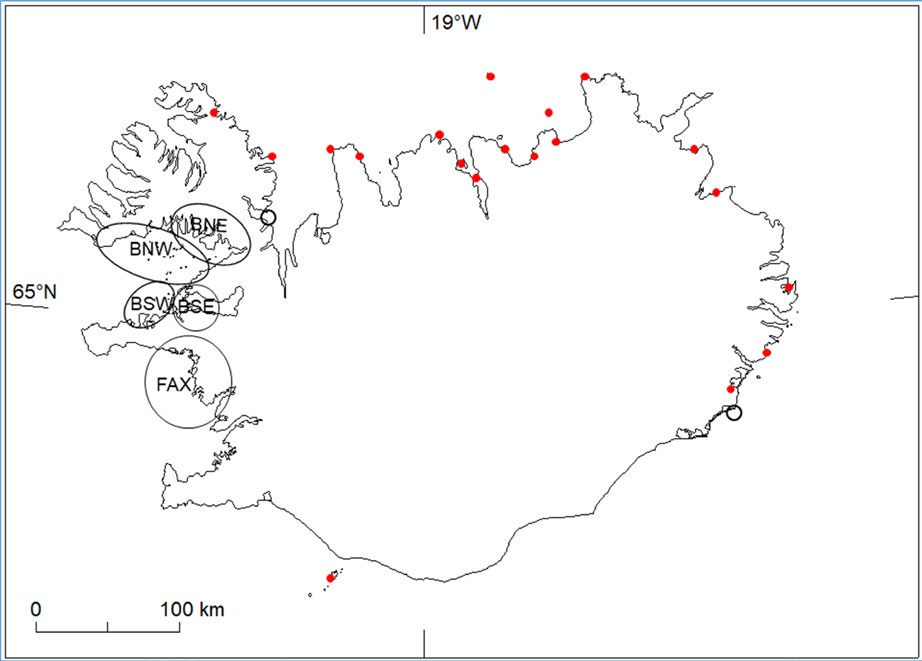
Map of Iceland showing the breeding distribution of Cormorant Phalacrocorax carbo. Black outlines show the present (1975–2015) breeding regions on the west coast: BNW (Breiðafjörður Northwest), BNE (Breiðafjörður Northeast), BSW (Breiðafjörður Southwest), BSE (Breiðafjörður southeast), FAX (Faxaflói). Red dots show abandoned colonies on the N, E and S coast recorded some time between 1780 and 1970. Unfilled circles show new colonies on the N and SE coasts

The Atlantic subspecies of great cormorant (hereafter cormorant) is a coastal seabird ranging across northern North‐Atlantic shores from northwestern Europe to west Greenland and eastern North America (Cramp & Simmons, 1977; Hatch, Brown, Hogan, & Morris, 2000). In Iceland, cormorants mostly occur in shallow coastal waters <20 m deep (Gardarsson, 2008). This habitat covers about 6,900 km^2^ unequally distributed along the coast of Iceland, with about half in the two west coast bays where almost all cormorants bred during the study period (Gardarsson & Jónsson, 2013), that is, Faxaflói and Breiðafjörður (Figure 2). The two bays are separated by the 90 km long and mountainous Snæfellsnes peninsula (20–25 km wide).

**Figure 3:**
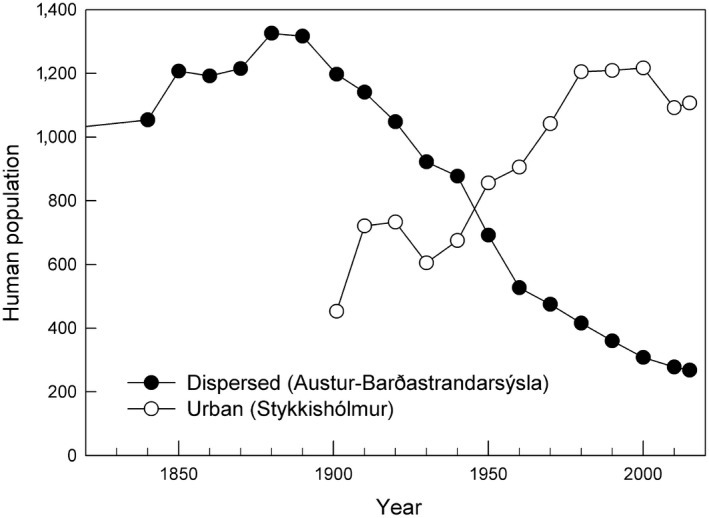
Censuses of two municipalities at Breiðafjörður on the west coast of Iceland in the period 1845 to 2015. The county Austur‐Barðastrandarsýsla, most of northern Breiðafjörður, (filled circles) was mainly occupied by dispersed farms and permanent or temporary fishing stations. The town Stykkishólmur, on the south coast of Breiðafjörður (unfilled circles), is a trade and fishing town. (Based on official census figures from Statistics Iceland)

Cormorants are large birds that seem to be of limited interest to avian predators but human exploitation of large colonial birds is widespread. In the low islets of Icelandic coasts, young cormorants were heavily exploited for meat. Early records suggest that, from the late 18th century until sometime in the early 20th century, the breeding distribution of cormorants was quite different from today, that is, colonies occurred mainly on the north and east coasts of Iceland (Faber, 1822; Hantzsch, 1905; Mohr, 1786). While low islets are numerous on the west coast, such islets are scarce elsewhere and cormorants breeding on north and east coasts (see Figure 2) nested mainly on coastal cliffs. During the 20th century, Icelandic society transformed from dispersed coastal lowland human occupancy based on a subsistence economy, into urban communities based at first on a large scale fishing industry. The industrial revolution shows up well in censuses of selected municipal units in Breiðafjörður in the last two centuries. The town Stykkishólmur conforms to a rise in the urban population during the 20th Century, whereas the rural Austur‐Barðastrandarsýsla shows the concurrent decline in the more dispersed farming communities (Figure 3). During this period, cormorants abandoned the historical north and east coast colonies and moved to the west, into the archipelagoes in Faxaflói and Breiðafjörður.

Coastal wildlife and associated natural resources, historically, were an important source of livelihood but their importance declined during the 20th century. Collection of eider down and eggs, along with sheep farming (livestock had to be tended and moved among islets), remain widespread activities but can cause disturbance to the wary cormorants which respond by nesting on islets shared by few other species and thus less attractive to humans. Boat traffic, often associated with fishing near the cormorant colonies, is also a potential source of human disturbance but we did not assess it. Physical catastrophes occur on rare occasions, most often attributable to coinciding high tides and high winds.

In July–September, many cormorants disperse from the west coast breeding colonies and through the winter they are found along the whole coastline of Iceland (6,000 km long). By March, the adults return to the breeding colonies and begin to build nests that last until July. The main laying period is in late April through May. Cormorants are migratory but generally remain within Icelandic waters; as of 2016, only one out of 363 ring recoveries (total 3,277 ringed) was reported from abroad, in the Faroe Isles some 400 km southeast of Iceland (Icelandic Institute of Natural History, *unpublished data*).

Cormorants presently are a legal quarry in Iceland. Traditionally, young were taken in the colonies but this harvest has declined almost to nothing in recent decades. In autumn and winter (September–March), cormorants are hunted along the coast and juveniles seem to be the preferred target. On average, 2047 (range = 1,362–3,336, *SE* = 127) cormorants were harvested annually in 1998–2015 (Iceland Environmental Agency, 2018); there was no annual trend in the harvest during the period (*R* ^2^ = 0.169).

### 2.2 Islets and colonies

Colony was our basic unit used for counting and recording breeding cormorants, defined topographically as a group of nests occupying an islet or a closely aggregated group of islets (within 100 m of each other) emerging from a common subtidal or intertidal shelf. Islets averaged 0.66 ± 0.11 (*SE*) ha in area (range 0.04–6.49 ha, *n* = 84), varied in soil cover but were devoid of woody vegetation. While each colony is topographically well defined and recorded as such, nests often moved between nearby islets from year to year within colonies (and probably among colonies also); this was rather obvious when the distance was small (e.g., <2 km) but probably could not be definitely detected at longer distances.

We grouped breeding islets according to soil cover and nesting seabirds into (a) rocky islets (or skerries) mean area 0.34 ± 0.05 ha (*SE*, *n* = 47) with almost no soil (<10% soil cover) and very few other colonially breeding seabirds except cormorants; and (b) grassy islets, mean area 1.07 ± 0.22 ha (*SE*, *n* = 37) with extensive soil (mean 38 ± 5% cover) and various breeding colonial seabirds, including the commercially valuable common eider (*Somateria mollissima*). We assume that human disturbance is likely to be minimal on the rocky islets (which are slippery and difficult to walk on) and more frequent on the grassy islets. Nests in one or more groups of nests on a single islet, or sometimes a tightly packed group of islets were recorded as separate colonies.

### 2.3 Breeding colonies and regions

The west coast shallow sea and islets are conveniently divided into five regions based on topography and benthic communities, one in Faxaflói (region FAX) and four in Breiðafjörður (Figure 2). The FAX colonies were on a narrow (mostly 1–4 km) offshore belt of islets stretching along the northeastern shore of the bay. FAX (886 km^2 ^<20 m deep) was mostly exposed sandy shallows with rocky ridges but also sheltered estuaries with extensive tidal mudflats and mussel beds. Relative to Breiðafjörður, there was little boat traffic in FAX. A total of 17 cormorant colonies were used in 1975–2015, 8 skerries and 9 grassy islets.

Breiðafjörður is a complex bay, subdivided into two branches (Gilsfjörður and Hvammsfjörður), with large shallow areas (2,915 km^2 ^<20 m deep) and numerous islets and islands. The exposed parts are more open to the ocean swell and mostly ice‐free in winter. Large areas are covered with coarse shell‐sand and turf‐ or crust‐forming algae and kelp forests are of limited extent (Gunnarsson, 1991). The inner sheltered regions have extensive kelp forests (which support relatively high densities of small fish, including young cod *(Gadus morhua)* and bullrout (shorthorn sculpin, *Myoxocephalus scorpius)*, see for instance Keats, Steele, & South, 1987, Stål, Pihl, & Wennhage, 2007) and can become ice‐covered in winter. These shallow areas of Breiðafjörður were divided topographically into four regions (Figure 2):

1. BSW (462 km^2^), a moderately exposed southwestern part with numerous small islets and a rather variable bottom. Ten colony sites were used, of which eight were skerries. BSW was mostly comprised of one group of colonies and there were small isolated colonies to the west and northeast.
2. BSE (378 km^2^), the southeastern inlet of Hvammsfjörður, separated from the outer bay by an archipelago of densely packed islands; much of this sheltered fjord has a deep (20–40 m) soft bottom. Rock‐lined channels with heavy tidal currents between the islands at its mouth support kelp stands. Fourteen colony sites were used, seven of which were skerries.
3. BNW (1,381 km^2^), an exposed western part, bounded on the east side by a line between Skarð harbor and Skálmarnes. BNW has mixed bottom, including extensive tracts of shell‐sand toward the northwest and some kelp stands in the south and east. Twenty‐seven colony sites were used, 22 of which were skerries.
4. BNE (694 km^2 ^<20 m deep), a generally sheltered northeastern part (Gilsfjörður), north of Skarð and east of Skálmarnes, with extensive kelp forests and a mixed bottom. Fourteen colony sites were used, nine of which were skerries.

### 2.4 Other relevant vertebrate species of the islands

The main marine mammals are harbor seal (*Phoca vitulina*) and gray seal (*Halichoerus grypus*), which potentially compete with cormorants for food. Animals that cause disturbance or depredate on seabird colonies, apart from man, include great black‐backed gull (*Larus marinus*) and glaucous gull (*L. hyperboreus*), Arctic fox (*Vulpes lagopus*), white‐tailed eagle (*Haliaeetus albicilla*), common raven (*Corvus corax*), and the introduced American mink (*Neovison vison*).

### 2.5 Census of breeding colonies

Aerial photographic censuses of all known cormorant colonies in Iceland were carried out in 1975, 1983–1984, 1989–1990 and annually 1994–2015, usually in mid‐May (Gardarsson, 2008; Gardarsson & Jónsson, 2013). The nest (usually, but not always, occupied) was the primary counting unit. Cormorant colonies were conspicuous from the air as white patches of bird excrement and were located and photographed using fixed‐wing aircraft flying at airspeeds of about 100 knots (180 km/hr). The exact location of each colony was recorded, using maps, aerial photographs, satellite images and differential global positioning system (dGPS). Aerial observations were supplemented by ground truth obtained from a variety of written records, as well as observations supplied by ornithologists and local inhabitants.

**Figure 4:**
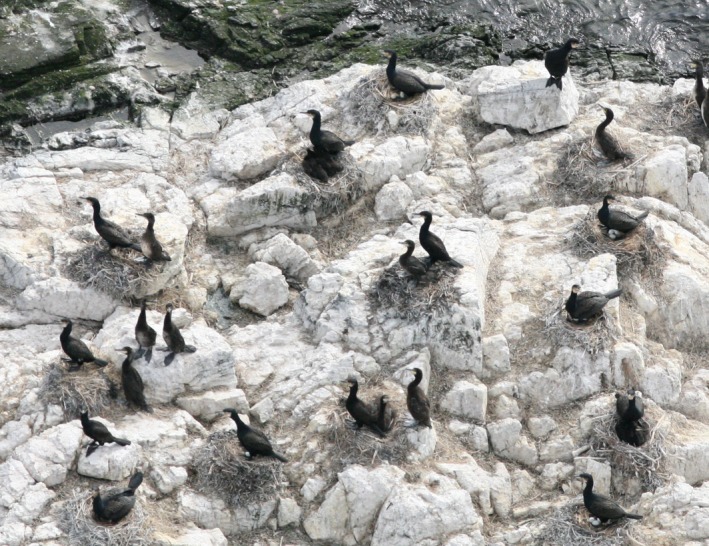
Nesting cormorants and broods at Akureyjarsker (BSE) in Breiðafjörður, West Iceland. Aerial photograph taken during a brood survey 20 June 2013. Note how the cormorants are conspicuous against the white backdrop. The white color comes from the bird's droppings and both allows easy detection of colonies from the air as well as providing a convenient background for nest counts from aerial photographs

Flight altitude (usually about 300–900 feet) and angle of view varied. Telephoto lenses (up to 300 mm) and a low angle of view were used for close views (Figure 4), for instance to distinguish cormorants from shags in mixed colonies and to estimate brood sizes. A high or vertical angle yielded better pictures for accurate nest counts. Medium format (55 × 55 mm picture frame) cameras with diapositive color film (slides) were used in 1975–2005 but were replaced by digital cameras in 2006. Films were placed under transparent acetate and counted in a stereoscope, marking each nest with a fine needle; digital images were counted in a computer using the program SigmaScan^®^.

### 2.6 Food of cormorants

The cormorant is a generalist feeder. In Iceland, the main food (about half the diet) in all seasons and places 1996–2000 was the bullrout, a noncommerical species abundant in the kelp forests. Other important cormorant foods were butterfish *Pholis gunnellus*, cod, saithe *Pollachius virens*, plaice *Pleuronectes platessa*, wolf–fish *Anarhichas lupus*, lumpsucker *Cyclopterus lumpus*, and the spider crab *Hyas araneus* (Lilliendahl & Sólmundsson, 2006). No abundance indices are available for the bullrout but the relative importance of each food fish may vary annually and prey choice probably depends more on fish size than species (Cech, Cech, Kubecka, Prchalova, & Drastík, 2008; Dias, Morais, Leopold, Campos, & Antunes, 2012; Gustavsen, 2017; Magath, Abraham, Helbing, & Thiel, 2016). We are not aware of any potential diet changes in cormorants during our study period 1975–2015.

**Figure 5:**
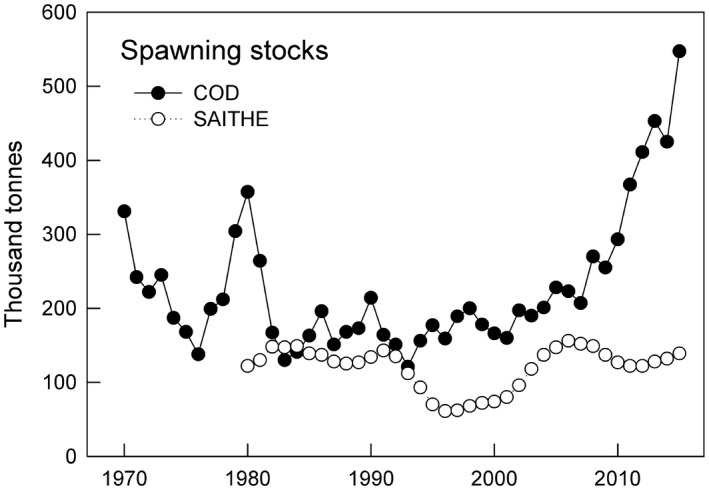
Spawning stocks of cod (*Gadus morhua*) and saithe (*Polacchius virens*) in Icelandic waters from 1970 to 2015 on (cod) and 1980 to 2015 (saithe). Data are annual estimates of spawning stocks of commercial fish, estimated annually by the Marine Research Institute of Iceland (2016)

The stocks of the commercial species cod and saithe have been monitored by the Marine Research Institute of Iceland (2016) annually since in the 1970's and we compared annual estimates of spawning stocks with total numbers of cormorant nests and number of juveniles. We used spawning stocks of cod and saithe as our indices of general fish abundances (Figure 5). This choice was based on: (a) a principal components analysis (PCA) to explore shared variation (59.8% with all loadings positive) among spawning stocks of cod, saithe, haddock (*Melanogrammus aeglefinus*) and common ling (*Molva molva*); (b) that cod and saithe are well known cormorant food items in the North‐Atlantic Ocean (Barrett, Røv, Loen, & Montevecchi, 1990; Gustavsen, 2017; Lilliendahl & Sólmundsson, 2006; Lorentsen, Grémillet, & Nymoen, 2004) whereas haddock and common ling were not listed among species eaten by cormorants in Iceland (Lilliendahl & Sólmundsson, 2006); and (c) the haddock and common ling indices added little informative variation to the PCA relative to those of cod and saithe, that is, common ling was highly correlated with cod (correlation matrix coefficient = 0.98) and haddock was highly correlated with saithe (correlation matrix coefficient = 0.77).

### 2.7 Cormorant brood size in late June

Breeding success is a potential indicator of local habitat quality as well as a convenient measure of fecundity. The great cormorant is notoriously shy when breeding, making it difficult to study breeding success at close range by visiting the colonies. Breeding success in large samples of nests was studied, in late June in seven years, 2007–2009 and 2012–2015, using low‐level aerial photography. When disturbed by the approaching aircraft, some cormorants left their nests but most incubating birds and those with small chicks stayed, completely covering the nests. When the young were half‐grown (or more) the parent could no longer cover them and it became possible to count the number of large young (Figure 4). Nest contents were classified (eggs, young of various size classes). The relative size of the young was estimated from that of the nearby attending parents. Small young, up to about one‐third size, could not be reliably counted; and thus, those were at least half‐grown and up to fully grown were used to estimate brood size. When the young reached full size, they began to wander away from the nest and to form crèches and thus became less countable again. During the brood surveys, an average of 66% (range 51%–81%, *n* = 10,852) of the nests contained countable broods.

### 2.8 Age composition in autumn and winter

In autumn (September) and in late winter (February), starting in 1998, age composition was surveyed (using spotting scopes) in the field in (a) southwest Iceland (several localities between Stokkseyri and Akranes), (b) at Snæfellsnes in west Iceland, and (c) in Húnaflói, north Iceland, mainly from Hólmavik to Vatnsnes. Study sites were selected on the basis of accessibility, distance and road connections. Most survey sites were situated outside breeding areas. About 3%–5% of the estimated total population was assigned to age class in each survey. In September, the proportion of juvenile cormorants was usually higher in the relatively accessible southwest than elsewhere in Iceland, leading to possible bias in the age composition. For the purpose of estimating age composition of the population in September and February, we used the geometrical means of two regions, southwest (survey area 1) and northwest (survey areas 2 and 3). In September, juveniles (pied brown with a variable amount of white below) were distinguished from adults (all black). In February, three categories were distinguished: juveniles (as before), adult nonbreeders (all black, without filoplumes), and full‐plumaged adult breeders (white thigh patches, white filoplumes on head and neck, nuchal crest) (Cramp & Simmons, 1977; Hatch et al., 2000; Figure 1). We assume that the number of adult breeders equals approximately two times the numbers of nests counted in May of the same year.

### 2.9 Climate indices

In the northern hemisphere, three main indices have been implicated as indicators of climate change: (a) an increasing trend in ambient and oceanic temperatures, often indexed by local T or SST, or by regional indices such as the Atlantic Multi‐decadal Oscillation index (Trenberth & Zhang, 2017), (b) changes in frequencies and occurrences of storms and prevailing wind conditions; in Europe, the North‐Atlantic Oscillation index (Hurrell, James, & National Center for Atmospheric Research Staff, 2016) often is used to explain changes in species abundances (Hátún et al., 2009); and (c) oceanic changes such as strength of the Subpolar Gyre reflect changes in the relevant ecosystems (Berx & Payne, 2017; Hátún et al., 2016). In addition to these climate indices, we used averaged monthly temperatures for January and February in Stykkishólmur (Icelandic Meteorological Office, 2018) as our local winter temperature index.

### 2.10 Statistical analyses

We evaluated annual trends in the data using linear regression. We compared correlations among regional nest numbers to test for spatial synchrony (using natural log (ln) of regional nest numbers) among our five regions (ten possible pairings) and applied false discovery rate (FDR) significance thresholds (which lowers *p*‐value thresholds below α  =  0.05, scaled with number of comparisons made) to consider spatial correlations simultaneously (Pike, 2011).

We used a generalized linear mixed model (PROC GLIMMIX, SAS Institute Inc., Cary, NC, USA) to evaluate relationships of weather and fish stocks to total nest numbers and proportion of juveniles in September, following an approach outlined in Jónsson, Lúðvíksson, and Kaller, (2017). This method includes variation due to year (autocorrelation) as a random effect in the model. Explanatory variables were climate indices SPG‐I, NAO, AMO, average winter temperatures for the months of January and February, and spawning stocks of cod and saithe. We used backwards model selection to identify the variables which were related to our dependent variables (total nest numbers and proportion of juveniles in September). Total nest numbers were analyzed at year lags 0–5 to estimate effects on recruitment into the breeding population because cormorants generally begin breeding 3–5 years old (Bregnballe, 2006; Cramp & Simmons, 1977; Frederiksen & Bregnballe, 2001; Janiszewski, Minias, Lesner, & Kaczmarek, 2017). We saw no biological reason to lag proportion of juveniles in September. As with the spatial correlations, we applied FDR a posteriori to the outcomes of the 36 tests (six explanatory variables and six time lags).

To compare effects of year and region on productivity, brood size data (2007, 2008, 2009 and 2012, 2013, 2014, 2015) were analyzed with a multivariate analysis of variance (MANOVA), with frequencies of brood sizes (1, 2, 3, 4 and 5) within a year (scaled by sample sizes) as response variable and year and region as explanatory variables. There were not enough degrees of freedom to test the year*region interaction. We report average brood sizes for these years; findings were the same between statistical tests on effects of year and region on average brood size and frequencies of brood sizes.

## 3 RESULTS

### 3.1 Total nest numbers

**Figure 6:**
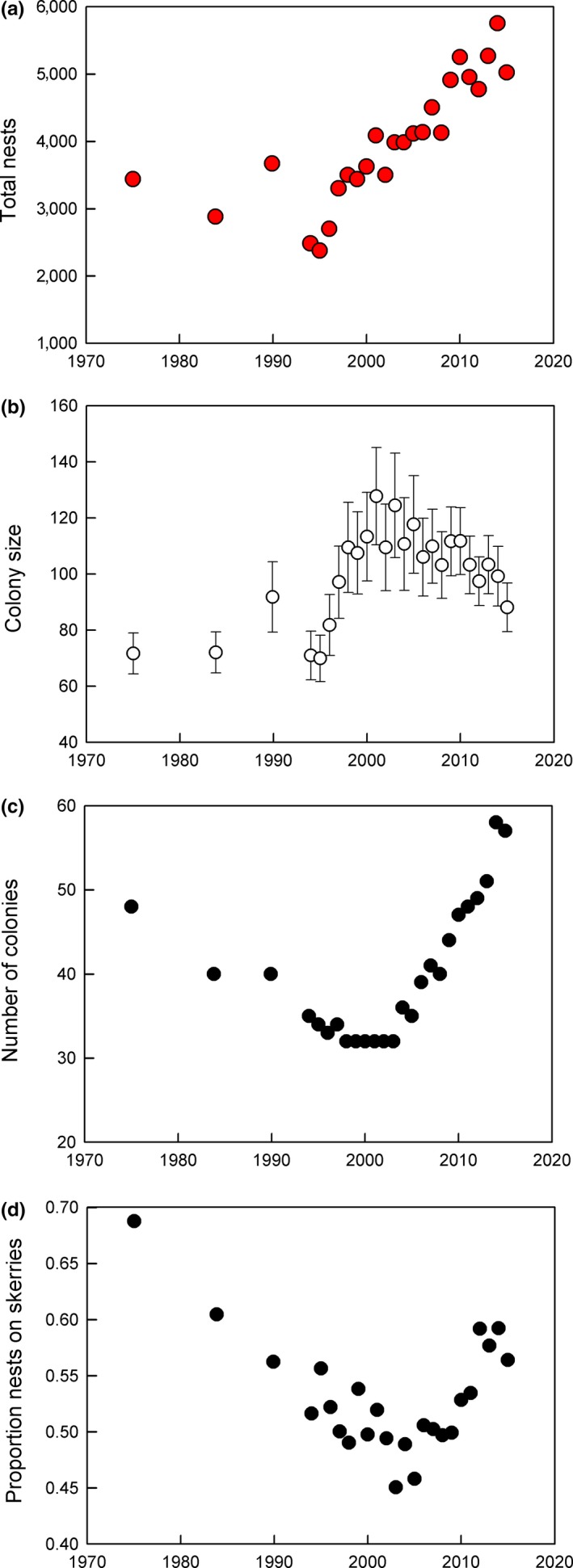
Total numbers of cormorant nests censused in Iceland during 1975 to 2015. (a) Total nest numbers. (b) Mean colony size ± *SE*. (c) Number of colonies found in each census. (d) Proportion nests on skerries (=rocky islets with <10% soil cover), see text for details

Nearly all breeding colonies were in two large bays on the west coast, the northern half of Faxaflói, with 17% of the cormorant nests in 1975 and 29% in 2015, and Breiðafjörður with 83% of the total in 1975 and 69% in 2015. Only three complete counts were available in 1975–1990, among which total numbers were approximately stable, around 3,000 nests (Figure 6a). When annual counts began in 1994, nest numbers had recently declined sharply in all five breeding regions. In 1995, there was an all‐time recorded low of 2,376 nests; after that numbers increased, reaching a high of 5,752 nests in 2014 (Figure 6a). During 1994–2015 the rate of increase was log‐linear and averaged 0.035 ± 0.003 *SE* per year (*r^2^ *= 0.88, *p* < 0.001). In the first few years, the rate of increase was higher, for example in 1994–2001 *r* was 0.076 ± 0.065 (*r^2^* = 0.90, *p* < 0.001).

In the first censuses (1975, 1983–1984, 1989–1990), there were about 40 colonies and the average number of nests in a colony was about 70. Average colony size increased steeply in 1994–2003 to 124 nests, followed by a slow decline to about 90 nests in 2015 (Figure 6b). Number of colonies ranged 32–36 in 1994–2005 but increased to an average of 42 colonies (range 39–47) in 2006–2010, and further increased to an average of 53 colonies (range 48–59) in 2011–2015 (Figure 6c). The habitat distribution of colonies changed during the same period, with almost 70% on rocky islets (skerries) in 1975, decreasing to 45% in 2003 and followed by an increase to about 60% by 2014 (Figure 6d).

### 3.2 Total nest numbers in relation to environmental variables

Backwards stepwise model selection indicated that cod and saithe indices were positively correlated to total nest numbers for 4 and 3 of 6 time lags, respectively (Table 1). SPG‐I was inversely correlated to total nest numbers for 3 of 6 time lags (Table 1). Effects of cod and SPG‐I were relatively immediate (lags 0–3 and 0–2) compared to effects of saithe which were more delayed (lags 3–5). NAO and local winter temperature were inversely correlated to total nests numbers for 1 lag each (Table 1).

**Table 1:** Final generalized mixed models from backwards stepwise model selections (PROC GLIMMIX) on total nest number of cormorant *Phalacrocorax carbo* in Iceland 1994–2015

Time lag	Index	Estimate	Num DF	Den DF	*F*	*p*
0	Gyre index	−615.9	1	19	31.5	0.0001
0	Cod	3.6	1	19	17.5	0.0005
1	Gyre index	−532.9	1	18	25.2	0.0001
1	Cod	4.3	1	18	17.7	0.0005
2	Winter temperature	−210.6	1	15	7.2	0.02
2	Gyre index	−472.7	1	15	14.3	0.002
2	Cod	5.1	1	15	22.8	0.0002
3	Saithe	7.2	1	15	7.4	0.02
3	Cod	4.2	1	15	11.4	0.004
4	NAO index	−76.8	1	14	7.0	0.02
4	Saithe	12.3	1	14	25.8	0.0002
5	Saithe	10.3	1	14	11.9	0.004
5	Cod	7.12	1	14	7.1	0.02

Note

Explanatory variables considered were indices of fish stocks (cod *Gadus morhua* and saithe *Pollachius virens*) and climate indices (Winter temperature, Subpolar gyre index, Atlantic multi‐decadal oscillation (AMO) index and North‐Atlantic Oscillation (NAO) index.

### 3.3 Regional numbers and spatial correlations

In 1975–2015, the patterns of change in numbers of nests were broadly related to regions. Spatial synchrony among regions was limited to region BSE, where nest numbers were positively correlated with those of: (a) BNE (*r* = 0.616, *p* = 0.007), (b) BNW (*r* = 0.591, *p* = 0.014), and (c) BSW (*r* = 0.577, *p* = 0.020), whereas nest numbers were not correlated among other regions (FDR‐adjusted *p*‐values > 0.08). Evidently, spatial correlations (3 of 10 pairings) were related to distance and geographical barriers and no correlations were found between FAX with any of the four regions in Breiðafjörður. Regional trends in total nest numbers did not directly reflect change in the whole metapopulation and were apparently influenced by regional factors.

### 3.4 Regional nest densities (km^2^ sea <20 m deep)

**Figure 7:**
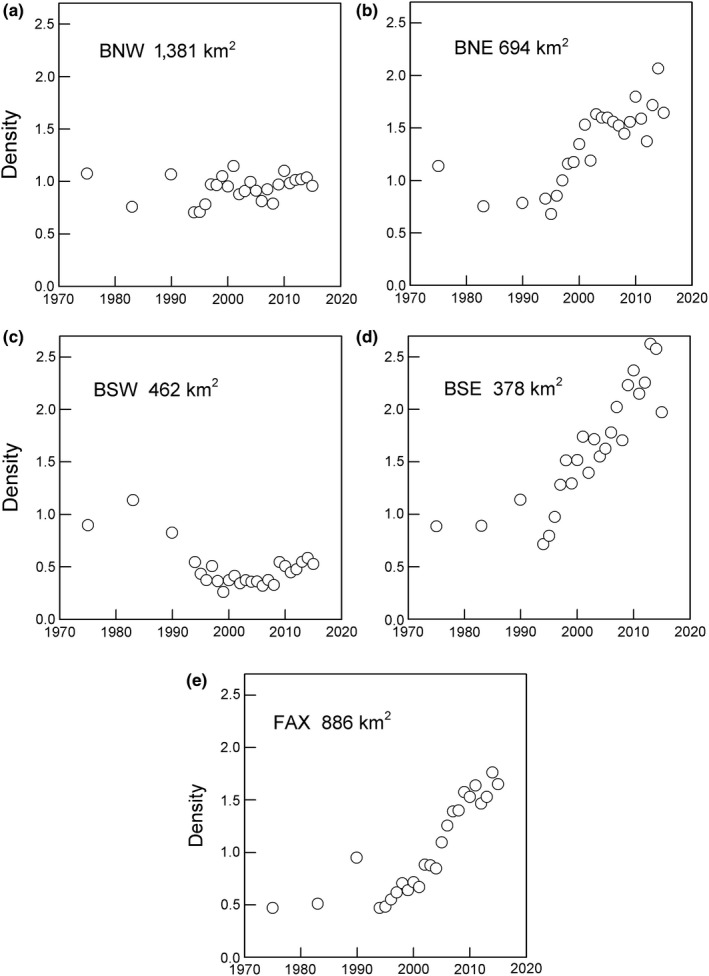
Densities of cormorant nests (numbers km^‐2^ < 20 m deep sea) in five regions on the west coast of Iceland. The variation among regions suggests that one region (panel a) was at carrying capacity for about 40 years, one (panel c) declined early and remained low whereas the other regions (panels b, d, and e) were able to receive immigrants during the population increase

In region BNW, the outer northwest Breiðafjörður, there was little change in nest density (0.94 ± 0.02/km^2^) during 1975 to 2015 (Figure 7a). In BNE, density decreased in 1975–1995 and then rapidly increased, *r* = 0.091 ± 0.016 (*r* ^2^ = 0.82, *p* < 0.001), to an asymptote, reached in 2003, of about 1.62 ± 0.05/km^2 ^(Figure 7b).

In BSW, the outer southwest Breiðafjörður, nest numbers developed differently from other regions and were relatively high (density about 0.8–1.1/km^2^) in 1975–1990 but then declined, and in 1994–2015 the mean density was 0.64 ± 0.03/km^2^ (Figure 7c). There was a decrease in 1994–2006 at an annual rate of −0.027 ± 0.012 (*r* ^2^ = 0.30, *p* = 0.051), followed by an increase of 0.051 ± 0.018 (*r* ^2^ = 0.52, *p* = 0.028).

In BSE, the inner sheltered part of southern Breiðafjörður (Figure 7d); annual increase followed a log‐linear curve with little annual variation. Densities in 1975–1989 were about 1 nest per km^2^. After a low of 0.68 in 1994, densities increased until 2015 at a mean annual rate of 0.071 ± 0.007 (*r* ^2^ = 0.84, *p* < 0.001) reaching a maximum of 2.5 nests per km^2^ in 2013 (Figure 7d).

In FAX, nest numbers were low in 1975. There was an increase in the 1980s, but by 1994 numbers had receded. Thereafter numbers increased at a mean annual rate of 0.071 ± 0.004 (*r^2^* = 0.93, *p* = 0.001), reaching a maximum of 1,560 nests (1.9/km^2^) in 2014 (Figure 7e). In FAX, maximum carrying capacity, as indicated by the asymptote in 2009–2015, was 1.59 nests per km^2^.

### 3.5 Brood size surveys in late June

**Figure 8:**
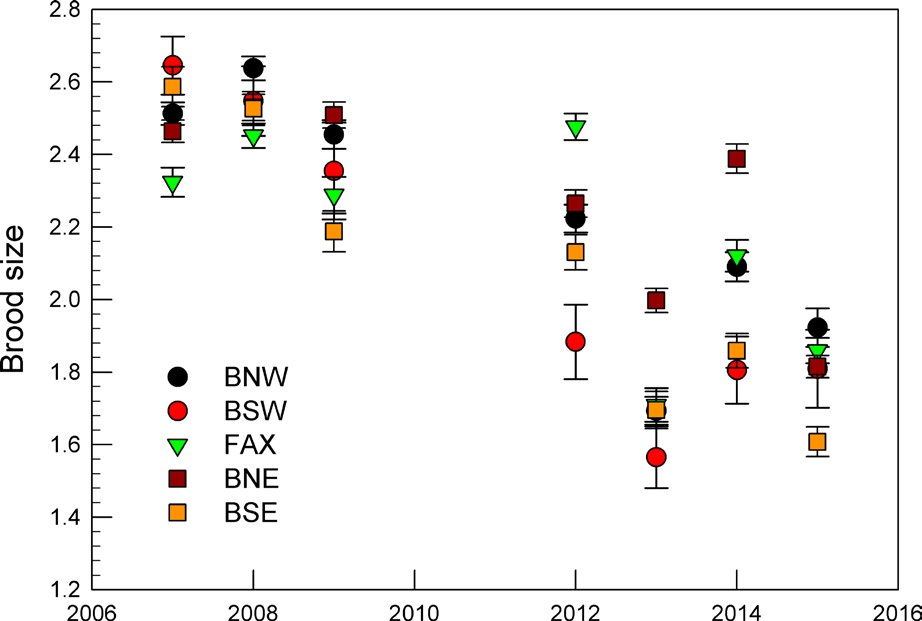
Mean brood sizes ± *SE* of cormorants in five regions on the west coast of Iceland. The decreased brood sizes in 2012–2015 (right side of panel) compared with 2007–2009 (left side of panel) coincide with a plateau in nest numbers (See Figure 5a), suggesting the first indication of density‐dependent limitation of breeding output

Brood size (i.e., the number of half‐full grown chicks in successful broods) was estimated in large samples of nests in seven years, 2007–2009 and 2012–2015 (Figure 8). Each year the average brood was similar among regions but there was a significant decline with the years (*r^2^* = 0.76, *p* = 0.011). In 2007–2009, the average brood was about 2.4 and very similar in all regions but declined to 1.8 in 2012–2015. MANOVA reported that brood sizes differed among years (Wilks' Lambda (WL): 0.02, *F*_30,82_ = 4.89, *p* = 0.0001) but not among regions (WL: 0.28, *F*_20,67_ = 1.6, *p* = 0.08).

### 3.6 Proportion of juveniles in September 1999–2014

**Figure 9:**
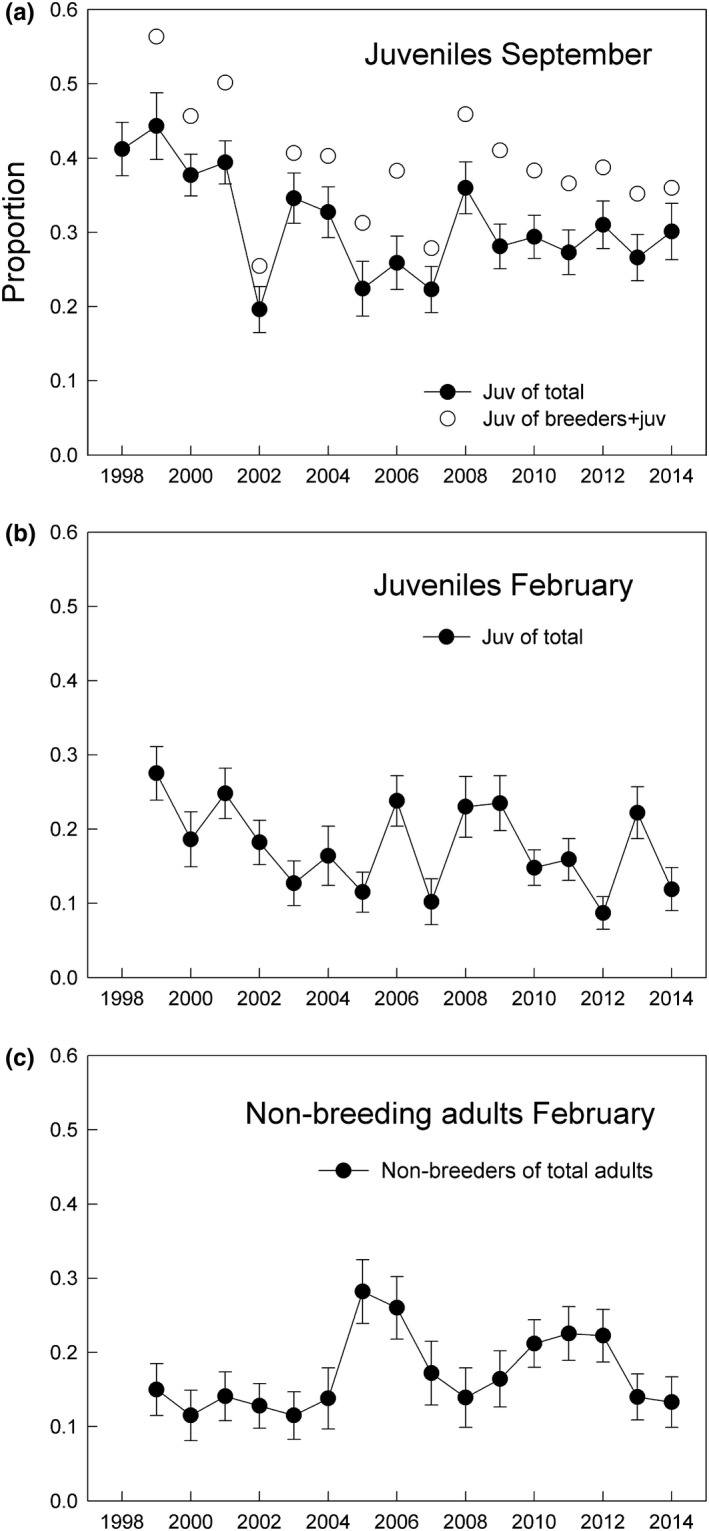
Age composition of cormorants was estimated from surveys on land September and February 1998–2014. In (a) the filled dots with the solid line show the observed proportion of juveniles in September, whereas the unfilled dots show the estimated proportion of juveniles in September corrected with estimates of breeding and nonbreeding adults in September. In February (b,c), three categories were distinguished: juveniles (as before), adult nonbreeders (all black, without filoplumes), and full‐plumaged adult breeders (white thigh patches, white filoplumes on head and neck, nuchal crest

**Figure 10:**
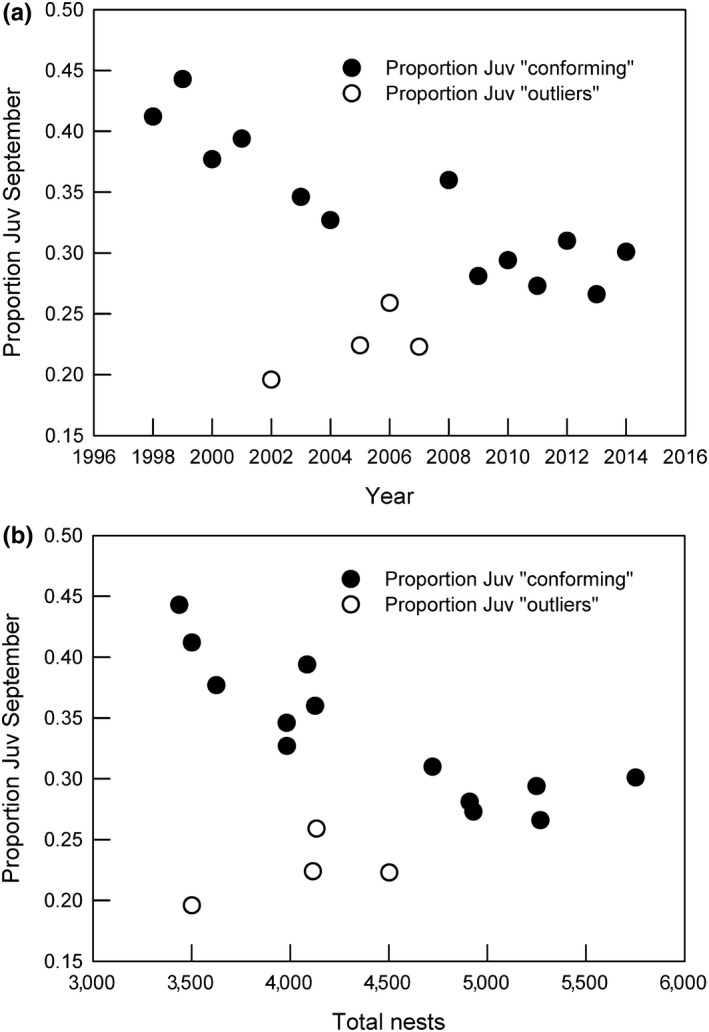
The proportion of juvenile cormorants in September 1998–2014 plotted against (a) year and (b) total nests. In (a) the relationship is statistically significant for the sum of conformers (to density dependence) and outliers (*b *= −0.0684, *F* = 4.875, *p* = 0.043, *R^2^* = 0.227) but for comformers alone *b *= −0.0684, *F* = 45.163, *p* < 0.001, *R^2^* = 0.804)). In (b) the relationships are weaker: for the sum of conformers and outliers *(b* = 0.00006,* F* = 2.601, *p* = 0.128, *R^2^* = 0.148). If four outliers are removed, an apparent density‐dependent relationship emerges (*b* = 0.000065, *F* = 32.387, *p* = <0.001, *R^2^* = 0.746).

In September, juvenile cormorants are distinguishable from older (1 year+) birds but breeding and nonbreeding adults look alike; and thus, the proportion of juveniles out of the total number of cormorants is therefore an underestimate of fecundity. The proportion of juveniles averaged about 0.31 and was generally higher on the SW coast (mean 0.45) than on the N coasts (mean 0.23). The geometrical mean proportion of juveniles declined during the study period from over 0.4 to about 0.3, with the lowest values in 2002 and 2005–2007 (Figure 9a). The proportion of juveniles declined with the years, and with density, suggesting weak density dependence, however the relationship of (Y) proportion of juveniles with (X) year was stronger than with (X) density. Also, there were four outliers (2002, and 2005–2007), which suggest that density per se was not limiting the production of juveniles and could be a result of more than one limiting resource, such as two or more demersal fishes for which stock models were not available. A suggestion of density dependence in the proportion of juveniles can be observed if 2002 is removed as an outlier, upon which a negative relationship with total numbers is found (Figure 10; *F* = 8.075, *r* = 0.520, *p* = 0.013).

In the southwest, the proportion of juveniles was usually about 0.5 in 1998–2004 and then declined, reaching a minimum of 0.27 in 2013, although high proportions were also found in 2008 and 2012. In the north, the proportion of juveniles was highest, about 0.38, in 1998 and 1999, declined to <0.1 in 2002 and was around 0.2 in 2008–2015. Backwards stepwise model selection indicated that fish stocks (cod and saithe) and climate indices (AMO, NAO, SPG‐I, and winter temperatures) were not correlated with the proportion and the estimated number of juveniles within the year (lag = 0).

### 3.7 Age composition in February 1999–2014

Three age groups could be distinguished in February (late winter). The proportion of juveniles (first‐year) was similar between the north and southwest coasts and also relatively similar among years, with high values of about 0.3 and low values about 0.1 (Figure 9b). The southwest coast averaged about 0.21 and the north coast about 0.17. The geometrical mean of the two regions was 0.18, varying between 0.09 and 0.28. The proportion of nonbreeders (subadults) was mostly a little lower along the north coast, mean about 0.16, than in the southwest, mean 0.20. Combining the regions, the proportion of nonbreeders averaged 0.17, with high values (around 0.27) reached in 2005–2006 (Figure 9c).

### 3.8 Estimates of annual survival of age classes

The proportional age composition in February was combined with the absolute number of breeding adults estimated from the nest numbers in May to yield a crude estimate of numbers of the three age classes and the annual survival of adult cormorants in 1999–2014, 0.850 ± 0.026 (Table 2). The calculated number of juveniles each September, compared with the estimated number of juveniles in the following February, yielded an estimate of average juvenile winter (September–February) survival of 0.471 ± 0.066 (*SE*) (Table 3).

**Table 2:** Estimated age composition of the Iceland cormorant population in 1999–2014

February	Adults			Juvenile		Sample sizes		(A + G)/Total(*t*−1) Annual adult survival
	Breeding	Nonbreeding	Total ad	Juv feb	Total			
	A	G	A + G	J	A + G+J	A, G	J, A	
1999	6,860	1,211		3,061	11,132	396	578	0.734
2000	7,234	940	8,174	1,868	10,042	342	425	0.946
2001	8,160	1,339	9,499	3,133	12,632	426	607	0.636
2002	7,004	1,028	8,032	1,787	9,819	484	618	0.914
2003	7,940	1,032	8,972	1,305	10,277	383	462	0.893
2004	7,910	1,266	9,176	1,800	10,976	268	327	1.039
2005	8,190	3,217	11,407	1,482	12,889	425	522	0.867
2006	8,268	2,905	11,173	3,490	14,663	418	604	0.742
2007	9,006	1,871	10,877	1,235	12,112	293	375	0.791
2008	8,254	1,333	9,587	2,864	12,450	289	398	0.944
2009	9,818	1,929	11,747	3,605	15,353	368	517	0.868
2010	10,500	2,825	13,325	2,315	15,640	619	838	0.818
2011	9,908	2,886	12,794	2,424	15,218	515	680	0.796
2012	9,420	2,698	12,118	1,150	13,268	531	652	0.924
2013	10,538	1,715	12,253	3,494	15,747	396	534	0.843
2014	11,504	1,765	13,269	1,791	15,060	385	472	–
2015	10,002	–	–	–	–	–	–	–

Note

Based on 2 x number of nests each May and observed samples in February. Also shown are estimates of adult annual survival February–February.

**Table 3:** Estimated winter survival of juveniles in the Iceland cormorant population in September‐February 1999–2005

	September numbers			February (*t* + 1)		
	Adults	Juveniles	Total	p Juv	Juv numbers	sJ Sep‐Feb
1999	9,539	7,587	17,126	0.443	3,061	0.246
2000	9,767	5,910	15,677	0.377	1,868	0.530
2001	10,073	6,549	16,622	0.394	3,133	0.273
2002	9,386	2,288	11,674	0.196	1,787	0.570
2003	9,711	5,138	14,849	0.346	1,305	0.350
2004	11,190	5,437	16,626	0.327	1,800	0.273
2005	12,000	3,464	15,464	0.224	1,482	1.007
2006	12,629	4,414	17,043	0.259	3,490	0.280
2007	10,776	3,093	13,868	0.223	1,235	0.926
2008	12,094	6,803	18,896	0.360	2,864	0.530
2009	14,303	6,366	20,669	0.308	3,605	0.364
2010	14,145	5,890	20,036	0.294	2,315	0.412
2011	13,580	5,101	18,680	0.273	2,424	0.225
2012	12,750	5,721	18,471	0.310	1,150	0.611
2013	14,455	5,247	19,702	0.266	3,494	0.341
2014	–	–	–	0.301	1,791	–

Note

Based on the calculated 6 months survival of adults (September‐February; cf. Table 2) and the observed proportion of juveniles in September.

**Figure 11:**
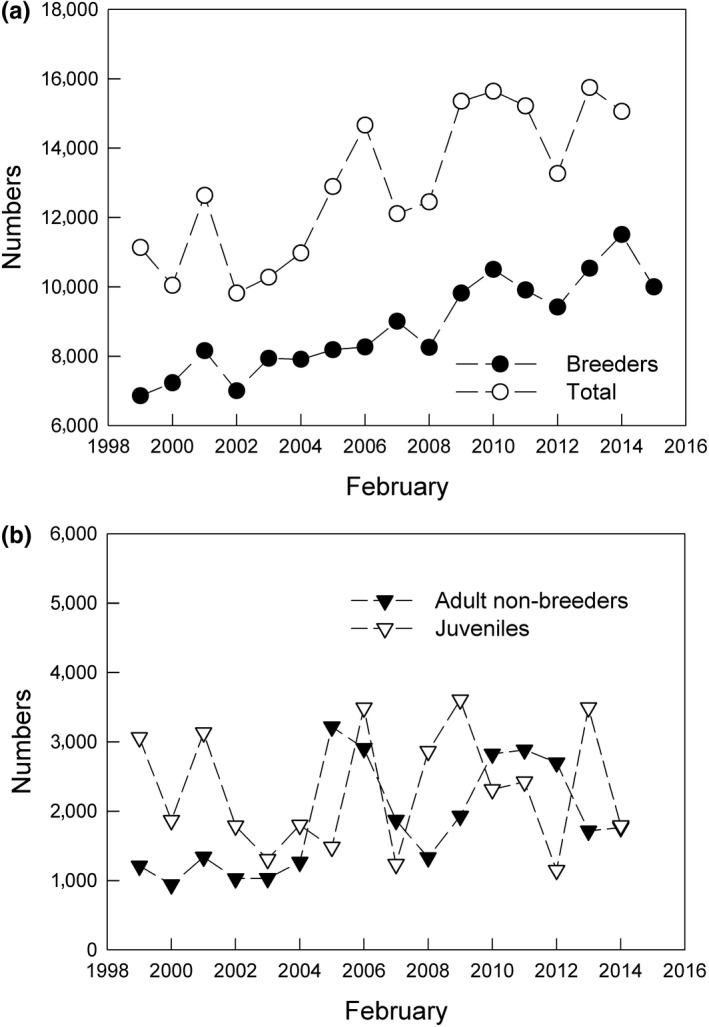
Estimated numbers (based on nest numbers and age composition in land surveys) of the cormorant metapopulation in Iceland in February 1999–2015. (a) Total numbers and number of adult breeders. (b) Number of nonbreeding adults and juveniles

Crude estimates of the total metapopulation, based on the three age groups distinguishable in the field in February combined with nest numbers, were used to explore the survival pattern of these groups through the period 1998–2015 (Figure 11). This comparison shows that the adult breeding population was steadily increasing through the period (as shown also by the unabated annual increase of 3.5% in overall nest numbers; Figure 11a).

## 4 DISCUSSION

Cormorants clearly enjoyed favorable conditions in 1995–2015 which were comprised of: (a) plenty of food, as indicated by spawning stocks; (b) favorable climate and oceanic conditions, and (c) reduced human disturbance, initially with abandonment of island farming in the 20th century and secondly with reduced interest in traditional harvest of eggs or wild birds in recent decades. Iceland is surrounded by some of the world's richest fishing grounds and these have since medieval times been the mainstay of the human population (Ólafsdóttir, Westfall, Edvardsson, & Pálsson, 2014). Human exploitation of the coast was for centuries characterized by dispersed farms subsisting by fishing from small boats, livestock farming and exploiting marine mammals and seabirds. Modern industrialized fisheries changed this way of life during the 20th century as shown clearly by official census records. Cormorants and other coastal birds have colonized new breeding locations as a result of humans deserting these dispersed coastal farms.

Cormorants, like many seabirds, were heavily exploited by man in the past and the population responded by altering its breeding habitat and geographical locations. A similar change in nesting habitat during the 20th century occurred with the white‐tailed sea‐eagle which switched from high cliffs to low coastal islets or hillocks, clearly in response to reduced persecution (Skarphéðinsson, 2003). This reduced exploitation of the coastal resources is likely to have affected the breeding cormorant population in recent decades although mostly before the beginning of the present study, which saw the end of an apparent shift in breeding distribution from the north and east coast to the west. This shift toward west coast shallow seas and islets to cormorant colonies probably corresponded to an ideal‐free distribution (Fretwell & Lucas, 1970; Røv, 1994). During such a response phase, first‐time breeders probably will seek out new colonies to avoid competition with experienced adults; once such recently undisturbed colony sites become occupied, dispersal is more likely to occur in an ideal‐free manner (Hénaux, Bregnballe, & Lebreton, 2007; Péron, Lebreton, & Crochet, 2010).

During our study, density of breeding regions varied strongly, apparently in response to local resources rather than changes in the total metapopulation. Thus, some form of local density dependence seems to limit breeding numbers at the regional scale (Frederiksen & Bregnballe, 2000; Røv, 1994), but the total breeding population is so far increasing steadily at the annual rate of 3.5%. This rate of increase is not shown by the young and nonbreeding sectors of the total metapopulation as estimated at the end of winter, suggesting that there may be another limitation to the numbers which may become more visible in the future, if the increase in nest numbers continues.

Cormorants need shallow waters (mainly less than 10m deep and as deep as about 20m; Debout, 1988) within reach of a secure place for resting and especially breeding. Shallow waters, <20 m deep, comprise about 6,900 km^2^ around Iceland, of which some 46% are along the coasts of Breiðafjörður and Faxaflói. The choice of islets as colony locations probably represents the optimal central place foraging sites (Christensen‐Dalsgaard et al., 2018; Sandvik et al., 2016; Shoji et al., 2015). Cliff sites, which were extensively used for colonies until in the mid‐20th century and are still used as roosts in the winter, may become used as islet habitat becomes fully occupied or (as presumably happened) islets in the best foraging habitat became unavailable due mainly to human disturbance.

### 4.1 Total nest numbers and changes in colony size and habitat distribution

The first census in 1975 and following estimates in 1983–1984 and 1989–1990 did not suggest major changes. An indication of declining numbers in the early 1990s led to the survey being carried out on an annual basis. Thus, the beginning of the annual phase of the study coincided with a population low in 1994–1995 which was followed by a major resurgence when total nest numbers increased by 3.5% p.a., with a maximum of 5,752 nests in 2014. This coincided with a shift from smaller to larger islets until about 2003, with a simultaneous decline in use of rocky islets (skerries), which suggests a causal link with changes in human disturbance. A possible explanation for this shift in nest site use may be changes in fish availability, caused either directly by numerical changes or by changed behavior of the fishes, as suggested by nest numbers in 1994–2015 being positively correlated with estimates of spawning stocks of cod and saithe.

An unexpected factor was that breeding densities were determined regionally, presumably by conditions in early spring (March–April) but brood size was independent of region, perhaps determined by fish supply to the small chicks. Brood sizes declined from about 2.5 in 2007–2009 to 1.8 in 2012–2015. This suggests that brood size was density‐dependent on a wide geographical scale; which fits with the important food fishes of the cormorant being widespread demersal species with planktonic larvae and similar shallow water habitat of the young. The juveniles and nonbreeding adults showed some annual variation in numbers but no trend, probably because they are presumably kept at low levels by intraspecific competition of the socially dominant adult breeders.

### 4.2 The role of climate change and its possible interaction with food

The shift in SPG‐I values from negative to positive in 1995 also coincided with the increased trend in total nest numbers after a low in 1991–1994. The negative trend in SPG‐I during this study period follows from an event in 1995 when the NAO and SPG‐I became uncoupled (Hátún et al., 2016), so it is conceivable that the generally favorable conditions for cormorants in Iceland stem from mild climate and improved feeding stocks, which in turn are associated with favorable oceanic conditions. A strong subpolar gyre (positive values) was associated with high adult survival in Brunnich's guillemot in Svalbard (Fluhr et al., 2017) but here we find the opposite effect in cormorants, where a weakened subpolar gyre is beneficial for food availability (fish stock indices) and concurrent metapopulation growth in great cormorants. We suggest that a weakened subpolar gyre in 1996–2015 (a period dominated by negative SPG‐I values) was part of beneficial conditions for fish recruitment in shallow waters (<20 m depth) or possibly smaller fish species, which in turn helped cormorant recruitment.

Although the bullrout is the most important food fish of cormorants in Iceland 1996–2000, several other species are taken (Lilliendahl & Sólmundsson, 2006). Cod, saithe and several other fish species use the shallow Breiðafjörður and Faxaflói as spawning and rearing grounds. Thus, we used available indices of the abundance of these commercially important fish species as a surrogate for availability of small fish. However, it is unlikely that cod and saithe were the single food‐related proximal causes.

### 4.3 Historical changes related to human activity prior to 1975

Almost all cormorant nests were found within Faxaflói and Breiðafjörður, with only two new colonies found outside this area (Húnaflói bay [northwest Iceland] established in 2010 and Lón [southeast Iceland] in 2015). In the 19th and early 20th centuries, there were few cormorant colonies on the islets of Breiðafjörður or Faxaflói (Faber, 1822; Hantzsch, 1905; Mohr, 1786), which were then colonized mainly in the 20th century. Before this study, there may have been a period of relatively high nest numbers in the 1950s followed by a low in the 1960s.

Historically, many cormorant colonies were found on the north, east and south coasts of Iceland (Figure 2). However, these breeding sites had already been abandoned by the onset of this study in 1975. At present, the cormorant population probably is in a transitional stage, that is, from a period of heavy exploitation and disturbance of breeding sites to a period of decreased human disturbance and markedly reduced harvest during the breeding season. Age composition is likely to change if the breeding population continues to increase.

### 4.4 Regional variation in nest densities

In 1975, 1983–1984 and 1989–1990, nest densities in all study regions were relatively low, mostly about 0.5–0.8/km^2^ shallow sea <20 m. The annual increase in total nest numbers of 3.5% in 1994–2015 was not equally distributed through the breeding range in western Iceland; each region maintained partly independent changes in nest densities. In Faxaflói (FAX) changes in nest density were not correlated with those in Breiðafjörður. In region BSW, nest densities declined from 1983 to 2008 when a slight increase (to 0.5) was noted. In the outer, most exposed Breiðafjörður region (BNW) most colonies increased during 1998 to about 2002 but after that densities levelled off or declined. Simultaneously, nest densities were increasing in the inner sheltered parts of Breiðafjörður (regions BNE + BSE). Asymptotic nest densities were generally higher in the sheltered (about 2 nests per km^2^) than the exposed regions (about 0.8 nests per km^2^). There are two possible explanations for this difference between outer and inner parts of Breiðafjörður:

1. The outer, more exposed regions are most likely subject to more variation in wave action which could lead to benthic upheavals, or perhaps biotic interactions within the epibenthic ecosystem, such as heavy grazing of benthic macroalgae by sea urchins *(Strongylocentrotus droebachiensis)* causing food resources in that area to deteriorate. Breeding numbers of another benthic/demersal feeding bird, the black guillemot, at Flatey in the outer Breiðafjörður (BNW) underwent a large fluctuation during the period 1975–2006, peaking in 1985–1993 and then decreasing (Petersen, 2001; A. Petersen, *personal communication*).
2. Differences in wave stress in the regions could also be expressed through historical changes in human exploitation of cormorants. We have very little direct information on the 19th ‐ early 20th century recolonization of the west coast by cormorants, during a time when the breeding islets were heavily exploited by the human population, who largely abandoned these islands in the 20th century. Presumably the outer exposed islets were less accessible to cormorant harvesters and the inner islets more accessible to humans using small open boats. The outermost islets were very important as fishing stations before the mid‐20th century and thus, were only colonized by cormorants shortly before the beginning of nest counts. Thus, it could be argued that the outer islets (BNW and BSW) were colonized earlier and by the time of this series of censuses, resources had become limiting in the exposed parts of Breiðafjörður but not yet in the sheltered parts (BNE and BSE).

The situation in the Faxaflói, which showed a continuing trend of increase during the study period, seems to lend support to both the explanations put forward above. The Faxaflói bay is more exposed to the ocean than the Breiðafjörður and thus probably more sensitive to benthic upheavals. There are previous records of large fluctuations in the numbers of cormorants nesting in Faxaflói (Gardarsson, 1996) and these could have been caused by fluctuating food resources.

## 5 CONCLUSION

At present, the total metapopulation increase of cormorant in west Iceland seems to be reaching carrying capacity, leading to colonization of other parts of the Icelandic coast, with no reduction in the rate of increase in the total breeding metapopulation. Simultaneously, the composition of the total metapopulation is likely to shift toward fewer floaters and perhaps juveniles and nonbreeding adults. We expect that further increases in this population will gradually become limited by available nest sites. However, historical colony sites around Iceland remain unoccupied and the reduction of cormorant harvest will allow many of those to become safe. New colony establishment occurs slowly but it is clearly the only way in which the present breeding population can increase further, because nest numbers at the regional level are limited by carrying capacity (nest density in relation to food resources). However, during the present study which has lasted 41 years, only two new colonies have been documented outside the continuous west coast range; presumably the expansion is restricted by behavioral constraints (especially philopatry) to remain near the natal or previous breeding colonies, or simply by dispersal distances; hence, most new colonies become established within the present range (see also Hénaux et al., 2007).

Finally, it seems likely that the age group structure of the Icelandic cormorant population will change as the total carrying capacity becomes more limiting, and that change in the level of small‐fish availability will continue to have a key role in limiting the population level.

## CONFLICT OF INTEREST

The authors declare no conflict of interest.

## AUTHOR CONTRIBUTIONS

Conceived and designed the study: AG. Analyzed the data: AG, JEJ. Drafted and wrote the paper: AG JEJ.

## Data Availability

Census and survey data: https://datadryad.org/. DOI: https://doi.org/10.5061/dryad.mt5q8m2. Data files: dryad‐data‐skarfur‐2019.
